# Medicinal utilization and nutritional properties of drumstick (*Moringa oleifera*)—A comprehensive review

**DOI:** 10.1002/fsn3.4139

**Published:** 2024-04-08

**Authors:** Zarina, Ab Waheed Wani, Monisha Rawat, Harjinder Kaur, Sachitanand Das, Taranpreet Kaur, Noor Akram, Zargham Faisal, Syed Saad Jan, Nabila Nusrat Oyshe, Mahbubur Rahman Khan, Yasir Abbas Shah

**Affiliations:** ^1^ Department of Horticulture, School of Agriculture Lovely Professional University Phagwara Punjab India; ^2^ Food Safety & Biotechnology Lab, Department of Food Science Government College University Faisalabad Faisalabad Pakistan; ^3^ Department of Human Nutrition and Dietetics Iqra University Karachi Karachi Pakistan; ^4^ Centre of Biotechnology and Microbiology University of Peshawar Peshawar Pakistan; ^5^ Department of Chemistry Hajee Mohammad Danesh Science and Technology University Dinajpur Bangladesh; ^6^ Department of Food Processing and Preservation Hajee Mohammad Danesh Science & Technology University Dinajpur Bangladesh; ^7^ Department of Food Science Government College University Faisalabad Pakistan

**Keywords:** anticancer, antidiabetic, miracle tree, *Moringa oleifera*, nutritional

## Abstract

The tropical and subtropical regions of the world support the growth of the Indian plant *Moringa oleifera*. It usually goes by the name drumstick tree or horseradish tree and thrives in warm climates. The leaves of the *M. oleifera* tree are now frequently used as nutrients and nutraceuticals due to their availability of various minerals. While having only very minor antinutritional effects, the leaves are abundant in many beneficial compounds. A recent review of the bioactive components and activity of moringa leaves has focused on both in vivo and in vitro studies. Drumstick leaves have antidiabetic qualities, anti‐inflammatory, anticancer, and antibacterial qualities among other health benefits. Phytochemicals, in addition to minerals and vitamins, are abundant in this vegetable. The majority of these effects, according to a review in the literature, are mostly brought on by the presence of carotenoids, glucosinolates, and phytochemicals. As a value‐added component in the production of wholesome meals, moringa is becoming more popular. Despite extensive research into locating and quantifying these advantageous elements in drumstick leaves, bioavailability and bioaccessibility studies were carried out. Beneficial photochemicals are absorbed and digested through incredibly intricate processes that involve several physicochemical and physiological interactions. Therefore, the biological impact of food may be attributed to its various metabolites that can access particular areas of action rather than its original substances. This body of literature offers the most recent findings in scientific research on the bioavailability, health advantages, nutritional profiles, and bioactive activities of moringa leaves as they relate to their use in a range of food products. Drumsticks are frequently used as a food element that promotes health because of their potent protection against a variety of ailments and the presence of environmental pollutants.

## Graphical abstract

This article provides an overview of moringa cultivation, its nutritional properties, and commercial and pharmacological uses. This body of literature offers the most recent findings in scientific research on the bioavailability, health advantages, nutritional profiles, and bioactive activities of moringa leaves as they relate to their use in a range of food products.

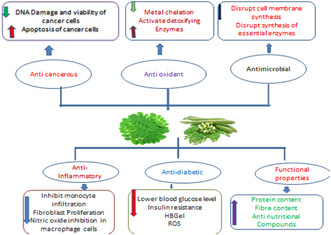

## 1 INTRODUCTION

The body's signaling channels might not always be the busiest ones (Anand et al., 2007). Beneficial photochemicals are absorbed and digested through incredibly intricate processes that involve several physicochemical and physiological interactions. Therefore, the biological impact of food may be attributed to its various metabolites that can access particular areas of action rather than its original substances (Mengucci et al., 2020; Selby‐Pham et al., 2017). Therefore, a deeper comprehension of the bioaccessibility, bioefficacy, and bioavailability of certain dietary components or nutraceuticals is necessary in order to establish compelling evidence for their efficacy. It is important to note that the human body views phytochemicals as xenobiotics, or compounds that are external to normal physiological function, and homeostasis and may be harmful if they are not digested and eliminated (Selby‐Pham et al., 2017). The stability and bioaccessibility of polyphenols from defatted *Moringa oleifera* seed flour, that is a by‐product of moringa oil, were assessed through an in vitro simulation of the human digestive tract. HPLC analyses demonstrated that the amount of free and bound phenolics increased significantly from salivary phase to gastric phase and subsequently to intestinal phase. From mouth to intestinal stage, 85.45% catechin, 80.04% epicatechin, 85.84% gallic acid, and 91% p‐coumaric were recovered in bound form (Swetha et al., 2018).

Research into medical plants and their benefits is constantly expanding due to therapeutic phytonutrients, which can hasten the creation of new drugs. Phenolic acids, flavonoids, carotenoids, saponins, tannins, and glucosinolates are just a few of the phytochemicals that are present in plants and have been shown to be beneficial for health and the prevention of cancer (Rockwood et al., 2013). Phytoconstituents, which are supplementary flavorful phytochemicals that prevent illness, are naturally occurring in plants. They are well known for helping to treat these conditions and lowering the chance of developing chronic diseases like cancer, cardiovascular disease, and neurological disease. A reliable treatment for malnutrition is *M. oleifera*, which is a member of the Moringaceae family. In all tropical and subtropical regions, *M. oleifera* thrives at a temperature of 25–35°C. In order for plants to flourish, the soil must be sandy or loamy, have a pH between 5.6 and 6.5, and receive a net rainfall between 250 and 3000 mm (Thurber & Fahey, 2010). In direct seeding, the seeds germinate quickly. The seed of moringa can be sown up to 2 cm deep in the soil and will germinate within 5–12 days. Containers can also be used to propagate moringa. Sand or loamy soil is placed in plastic bags with the saplings. A transplant can be done once it reaches a height of about 30 cm. Due to the delicate nature of tap roots, transplantation often damages them so utmost care must be taken when transplanting. In addition to cuttings with 1 m length and 4–5 cm diameter, the tree may also be grown from root cuttings, but these plants may not have deep roots. Drought and wind are common problems for such plants. The plantation of moringa on a large scale may be carried out intensively and semi‐intensively for commercial purposes. There is a slight difference in nutrients in trees grown in Nigeria and in trees grown in India. The nutritional differences between semideciduous and Savannah leaves were evaluated by (Asante et al., 2014). According to the study, the Savannah regions are mostly prone to high temperatures, resulting in less nutritional wheat. The difference in nutrient content could be due to denatured proteins and enzymes at higher temperatures. Soil plays a major role in determining plant nutrition and strength. According to (Dania et al., 2014), fertilizers can influence the nutrient composition of plant parts, regardless of whether they are used alone or combined. In a test comparing poultry manure, organic base fertilizer, and NPK fertilizer, poultry waste outperformed the other fertilizers for phosphorous, potassium, salt, and manganese. In addition, moringa stem girth has increased as has its vegetative growth when poultry manure is applied. Although nutrient variation exists in the plant, the overall nutrient profile remains the same. Moringa leaves, pods, seeds, and seeds contain many phytochemicals that make them one of the richest sources of nutrition. In particular, the leaves retain their nutritional value whether they are eaten raw, roasted, or dried and stored for a long time. Due to the presence of naturally occurring compounds such as flavonoids, phenolic acids, carotenoids, and glucosinolates, the leaves of this plant have possible applications as food supplements and health supplements in addition to being a source of nourishment (Berkovich et al., 2013; Mbikay, 2012). The main phytochemicals found in moringa leaves are astragalin, isoquercetin, and crypto‐chlorogenic acid, which are well known for their antioxidant, antihypertensive, and anti‐inflammatory properties (Oduro et al., 2008; Sánchez‐Machado et al., 2010). Based on the phytoconstituents and their antioxidant potential, several in vitro investigations have thoroughly substantiated the medicinal qualities and functional properties of this plant's extract (Barminas et al., 1998; Fuglie, 2005; Mbikay, 2012). Its strong antioxidant properties are mostly due to its high phenol content. Due to these health advantages, a number of pharmaceutical formulations made from this plant have been developed and are available in both the domestic and global markets (Lalas & Tsaknis, 2002; Yang et al., 2006). Researchers have shown that it has 4 times as much calcium as bananas, 7 times as much vitamin C as an orange, and 10 times as much vitamin A as a carrot. In addition to its easy cultivation, moringa can be used as an effective remedy for malnutrition. Children are treated with moringa in countries such as Senegal and Benin (Kasolo et al., 2010). When breast milk is not provided to children, they often show signs of malnutrition. In general, lactating mothers are prescribed lactogogues in order to increase milk production. As a precursor to the hormones required to promote reproduction, lactogogue is made of phytosterols. Moringa is considered an excellent source of hormone precursors due to its phytosterol content, such as stigmasterol, sitosterol, and kampesterol. Compounds like these activate estrogen receptors in the mammary gland, resulting in the proliferation of milk‐producing ducts. It is used to treat malnutrition in children younger than 3 years old (Mutiara Titi & Estiasih, 2013). Pregnant women need about six spoonsful of leaf powder a day to meet their iron and calcium needs. Providing an overview of moringa cultivation, nutrition, and medicine for commercial use, along with pharmacological properties, is the purpose of this study. Moringa has not been extensively studied for its use in treating diabetes and cancer. In the world today, it is possible to use moringa as a nutraceutical product.

## 2 MORPHOLOGICAL CHARACTERISTICS AND BIOGEOGRAPHICAL DISTRIBUTION

Moringa, a member of the Moringaceae family, is characterized by its fast‐growing nature and deciduous habit. It typically features compound leaves with multiple ovate or elliptical leaflets and produces small, fragrant flowers arranged in clusters. The elongated, cylindrical pods, known as “drumsticks,” contain dark‐brown seeds surrounded by fibrous pulp. This versatile tree exhibits adaptability to various environmental conditions, making it a valuable resource in tropical and subtropical regions worldwide (Leone et al., 2015). It is a swiftly growing deciduous tree that originates from the sub‐Himalayan regions of Northern India. Among the 13 species within its genus, *M. oleifera* stands out as the most widely distributed, particularly in tropical and subtropical regions with altitudes reaching up to 2000 m (Emmanuel et al., 2011). Presently, *M. oleifera* is predominantly observed in the Middle East, as well as in various countries across Africa and Asia. However, owing to its remarkable adaptability, it is progressively extending its presence to other regions, particularly tropical and subtropical areas affected by drought (Heuzé et al., 2016).

## 3 NUTRITIVE PROPERTIES

Because of its superior health and nutritional value, and positive benefits on the environment, *M. oleifera* is also known as the “Miracle Tree” or “Tree of Life.” All parts of the plant contain nutrients and antinutrients. The leaves of *M. oleifera* contain several minerals, including Ca, K, Zn, Mg, Fe, and Cu (Oduro et al., 2008). Additionally, beta‐carotene, pyridoxine, nicotinic acid, and folic acid are present in beta‐carotene as well as vitamins C, D, and E (Asiedu‐Gyekye et al., 2014). Furthermore, glucosinolates, isothiocyanates, glycosides, and glycerol‐1‐9‐octadecanoate are anticarcinogenic compounds, alongside tannins, sterols, terpenoids, flavonoids, saponins, anthracenediones, and anthraquinones (Wright et al., 2006). A diet containing moringa leaves can help reduce obesity because they have a low calorific value. Colon cancer can be prevented and treated with fiber‐rich pods (Oduro et al., 2008). There is an estimated 46.78% fiber content in immature pods, as well as a protein content of around 20.66% in the pods. In the pods, there are 30% amino acids; in the leaves, there are 44%; and in the flowers, there are 31% amino acids. There was no difference between immature pods and flowers in palmitic, linolenic, linoleic, and linoleic acids (Ijarotimi et al., 2013). Among the many minerals that contribute to the growth of moringa, calcium is one of the most important. Moringa leaves can provide 1000 mg of calcium, while moringa powder can provide 4000 mg, which is over twice as much calcium as 8 ounces of milk. Anemia can be treated with moringa powder, which can replace iron tablets. Moringa leaf powder contains 28 mg of iron compared with beef's 2 mg. Iron can be found in greater amounts in moringa than in spinach, according to a recent study (Asiedu‐Gyekye et al., 2014). It is essential to consume zinc in sufficient quantities in order to maintain the proper growth of sperm cells and to synthesize DNA and RNA. The amount of zinc per kilogram in *M. oleifera* leaves ranges between 25.5 and 31.03 mg, which is around the amount of zinc a person needs daily (Wright et al., 2006). They are rich in polyunsaturated fatty acids that regulate cholesterol levels, such as linoleic, linolenic, and oleic acids. Among these PUFA, the highest concentration of unsaturated fatty acids is alpha‐linolenic acid (Ijarotimi et al., 2013). In a recent study, moringa seed oil was found to contain approximately 76% PUFA, making it an ideal substitute for olive oil (Lalas & Tsaknis, 2002). Hot water extraction was recently used to isolate MOP‐2, a novel polysaccharide from moringa leaves. Several chromatographic methods have been employed to purify MOP‐2. Some functional foods may utilize this MOP‐2 as an immunoregulatory agent (Asiedu‐Gyekye et al., 2014). The body's vitality and several cardiovascular systems depend on moringa leaves, which are also rich in polyunsaturated fatty acids like omega‐3 and omega‐6. Moreover, it has fewer saturated fatty acids and more monounsaturated fatty acids. The content of nutrients changes depending on the region. According to Fuglie, the nutritional composition is influenced by the seasons (Asiedu‐Gyekye et al., 2014). Together with fat‐soluble vitamins like vitamin C and vitamin A (the precursor to beta‐carotene), *M. oleifera* also includes water‐soluble vitamin B complexes like folate, pyridoxine, and nicotinic acid (Wright et al., 2006). Approximately 252% and 235% of daily vitamin A and ascorbic acid needs are met by leaves of drumstick, respectively. Malnourished youngsters who received 10 g of dry leaf powder of moringa daily reported considerably more weight gain and showed signs of recovery much faster than the control group. The phytochemicals found in *M. oleifera* leaves, such as flavonoids, alkaloids, tannins, phenolic acids, and saponins, are a good source of the plant's anticancerous properties (Kaneto et al., 1999). It was discovered that vitamin A was in abundance during the hot, humid season, whereas vitamin C and iron were in abundance during the cool, dry season (Yang et al., 2006). The nutritional value of trees is significantly influenced by environmental factors in addition to location and climate (Moyo et al., 2011). Trans‐zeaxanthin (about 6%), trans‐lutein (about 30%), and trans‐b‐carotene (about 18%) comprise the large levels of carotenoids present in fresh leaves (Prentki & Nolan, 2006). Leafy greens contain significant levels of carotenoids, as well as tocopherol (36.9 mg/100 g) and vitamin C (271 mg/100 g). Phytates, tannins, saponins, and oxalates are just a few of the minor antinutrients found in the nutrient composition of *M. oleifera* (Kamalakkannan & Prince, 2006). They are neither toxic nor dangerous. When used in high doses, they may impede the consumption of several supplements, such as Zn, Fe, Ca, and Mg. Compared to the seeds and leaves of the bulk of legumes, including soybean, less phytate and saponin is present in its seeds and leaves. These factors make eating leaves safer and more nutrient dense (Aronson & Rayfield, 2002; Chumark et al., 2008).

## 4 BIOACTIVE CHARACTERIZATION OF LEAVES OF DRUMSTICK

**FIGURE 1:**
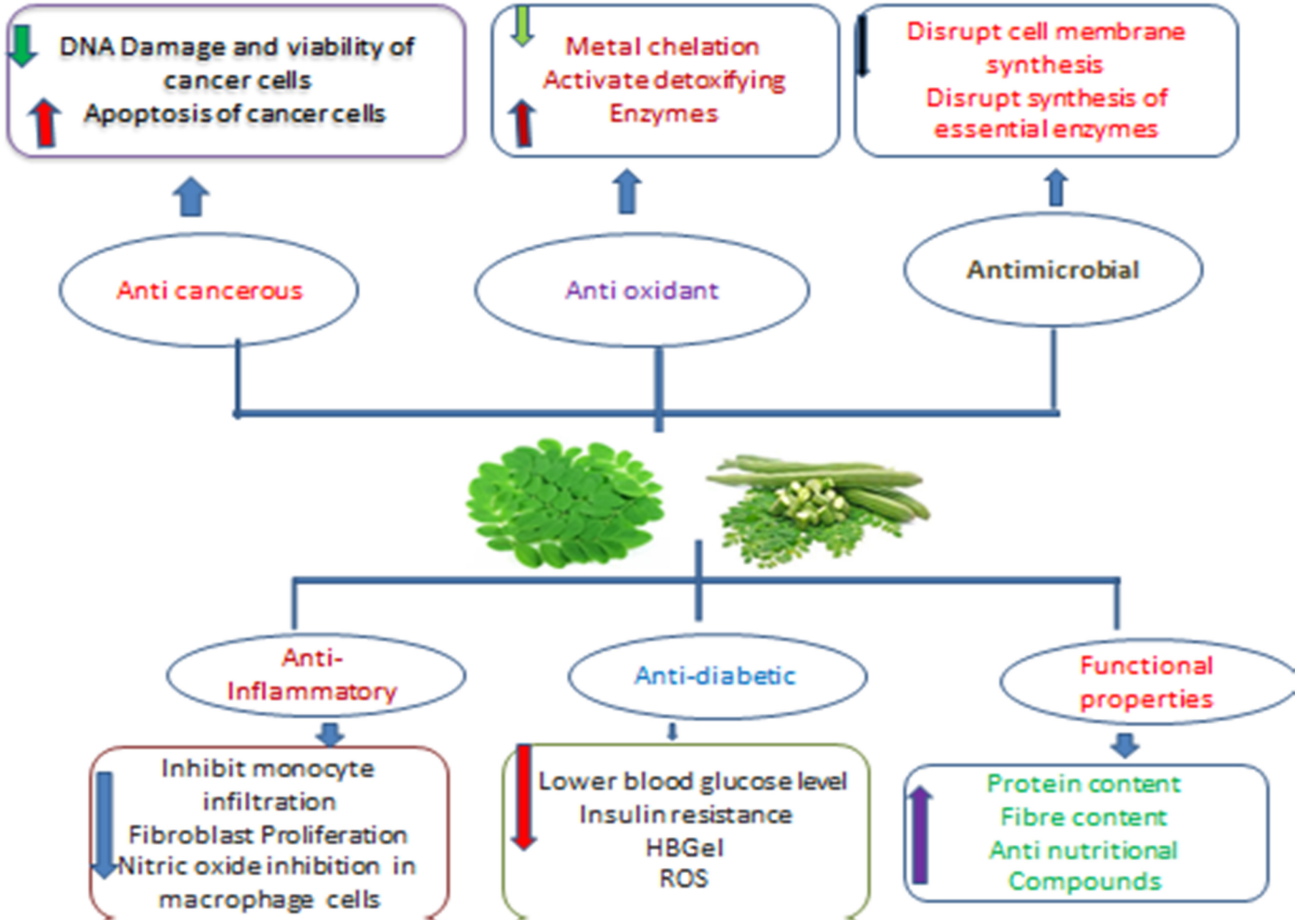
Medicinal benefits of drumstick leaves based on their bioactive features.

Plants include a variety of chemical substances that are used in food products and are physiologically active, such as isothiocyanates, phenolic acids, tannins, saponins, and flavonoids. These substances either have therapeutic activity or do not. Plants produce them to defend against physiological and environmental stressors such as microbial invasion and UV radiation (Jung, 2014; Tiloke et al., 2013). The leaves of the significant plant drumstick contain a variety of bioactive substances, including saponins, tannins, flavonoids, anthraquinones, catechol tannins, and alkaloids (Figure 3a–c) and as mentioned in Tables 1, 2, 3. These qualities make drumstick leaves useful for therapeutic and nutritional purposes and as water purifier. Moreover, Figure 1 shows the medicinal properties of drumstick leaves.

**TABLE 1:** Bioactive components in moringa leaves.

S. No.	Bioactive compounds	Amount	Molecular formula	Molecular weight
1	Vitamin A	1.28 mg	C_20_H_30_O	286.45
2	Vitamin B1	0.06 mg	C_12_H_17_ClN_4_OS	265.36 g/mol
3	Vitamin B2	0.05 mg	C_17_H_20_N_4_O_6_	376.36 g/mol
4	Vitamin B3	0.8 mg	C_6_H_5_NO	123.10 g/mol
5	Vitamin C	220 mg	C_6_H_8_O_6_	176.12 g/mol
6	Vitamin E	448 mg	C_29_H_50_O_2_	430.71 g/mol
7	Chlorophyll	80 mg	C_55_H_70_MgN_4_O_6_	907.4725 g/mol
8	Arginine	6%	C_6_H_14_N_4_O_2_	174.20 g/mol
9	Histidine	2.1%	C_6_H_9_N_3_O_2_	155.16 g/mol
10	Lysine	4.3%	C_6_H_14_N_2_O_2_	146.19 g/mol
11	Tryptophan	1.9%	C_11_H_12_N_2_O_2_	204.23 g/mol
12	Phenylalanine	6.4%	C_9_H_11_NO_2_	165.19 g/mol
13	Methionine	2%	C_5_H_11_NO_2_S	149.21 g/mol
14	Threonine	4.9%	C_4_H_9_NO_3_	119.12 g/mol
15	Valine	7.1%	C_5_H_11_NO_2_	117.15 g/mol

**TABLE 2:** Chemical structures of bioactive compounds present in moringa leaves.

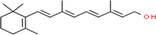 Vitamin A	Vitamin B1	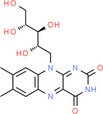 Vitamin B2
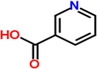 Vitamin B3	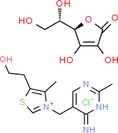 Vitamin C	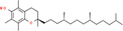 Vitamin E
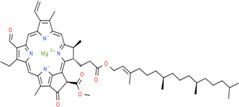 Chlorophyll	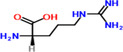 Arginine	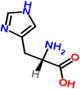 Histidine
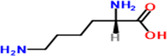 Lysine	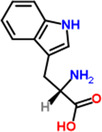 Tryptophan	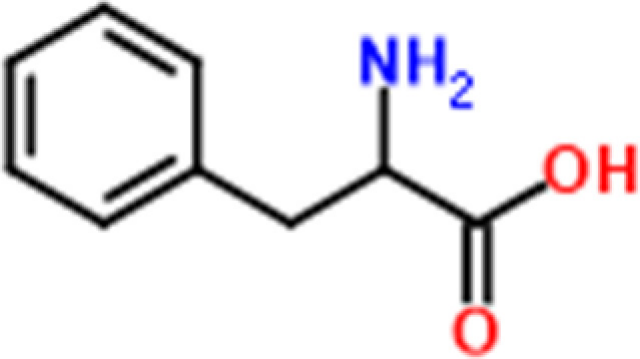 Phenylalanine
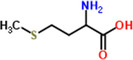 Methionine	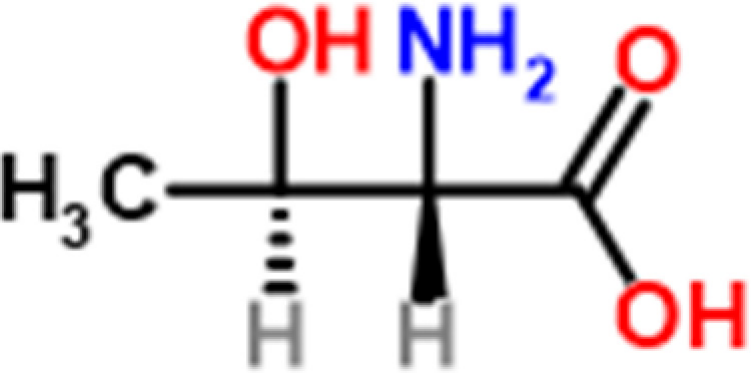 Threonine	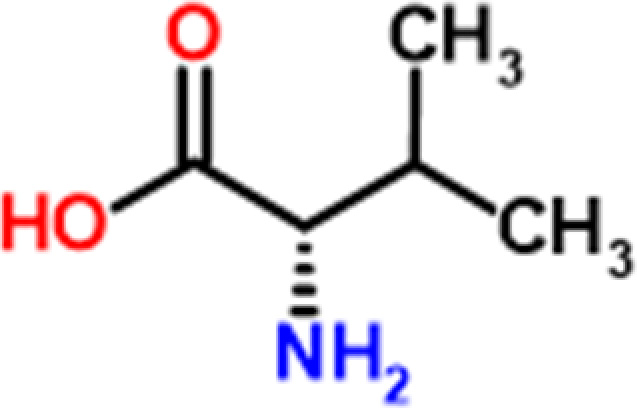 Valine

**TABLE 3:** Phytochemicals and biological properties of *Moringa oleifera*.

Plant part	Bioactive compound	Phytoconstituents	Chemical structure	Biological activity	References
Leaves	Niazirinin Niazirin Niaziminin Niazimicin A Niazimicin B	Benzyl Isothiocyanate	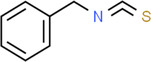	Anticancerous Antioxidant Antihypertensive Anticonvulsant Antibacterial	Milla et al. (2021)
Flowers	Quercetin Isoquercitin Kaempferitin Kaemopherol	4‐(Alpha‐L‐ rhamnopyranosyloxy) benzylglucosinolate	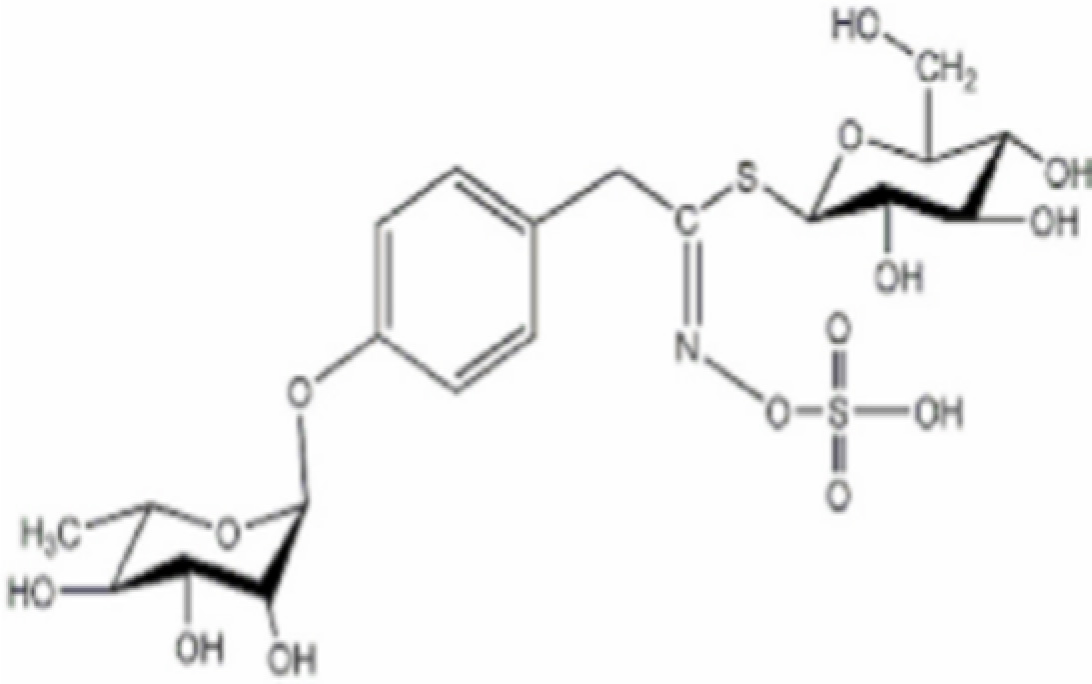	Works against inflammation	Miller et al. (2018)
Bark	Benzylglucosinolate derivatives	Pterygospermin	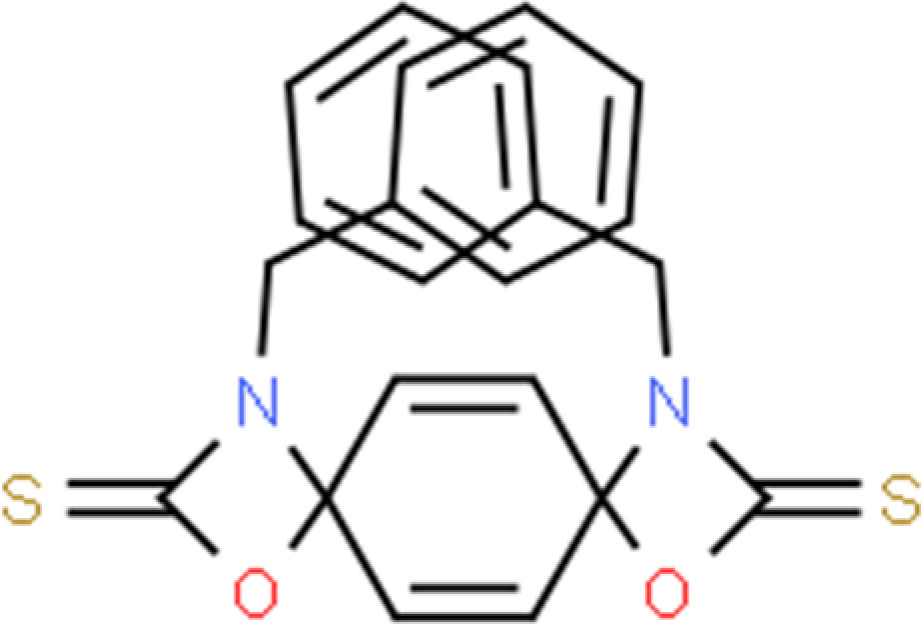	Acts against urolithiasis	Arora and Arora (2021)
Stem	Beta carotene Vanillin	Vanillin	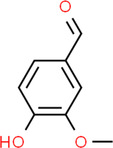	Works against inflammation	Balakumbahan et al. (2020)
Roots	Spirachin Moringinine Moringine p‐Cymene	Spirachin	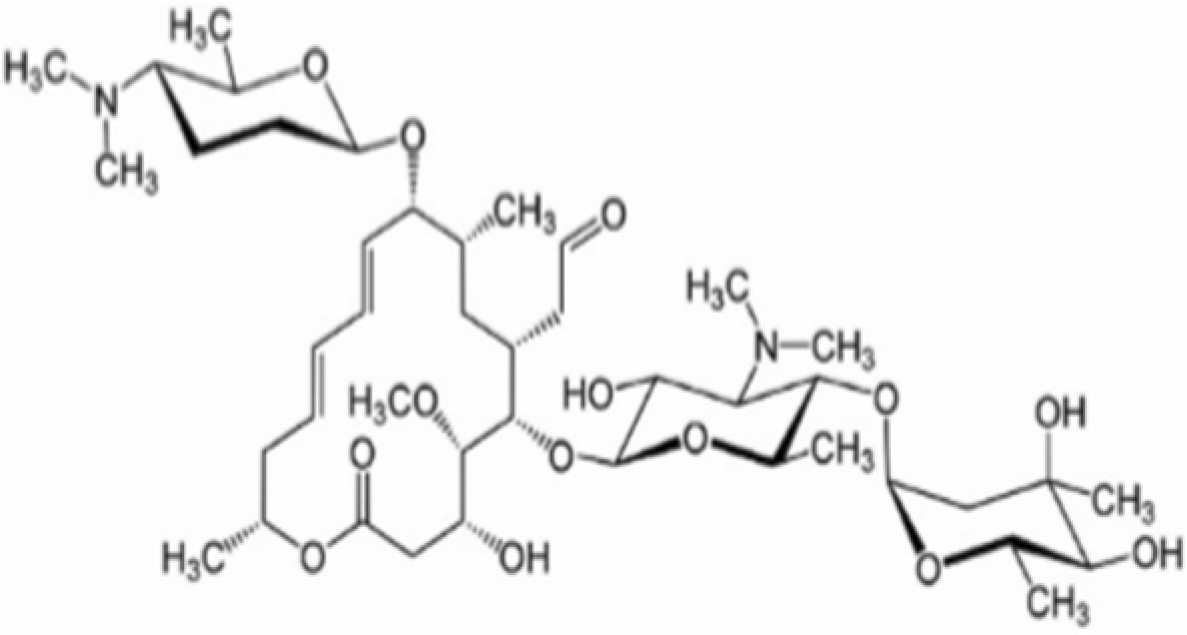	Antifertility	Srivastava et al. (2023)
Seeds	Niazimicin Niazirin Moringine	Niazimicin	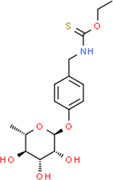	Acts against asthma	Saucedo‐Pompa et al. (2018)
Pods	Isothiocyanae Nitrites beta sitosterol	4‐(4‐O‐Acetylalpha‐L‐rhamnosyloxy) Benzyl isothiocyanate	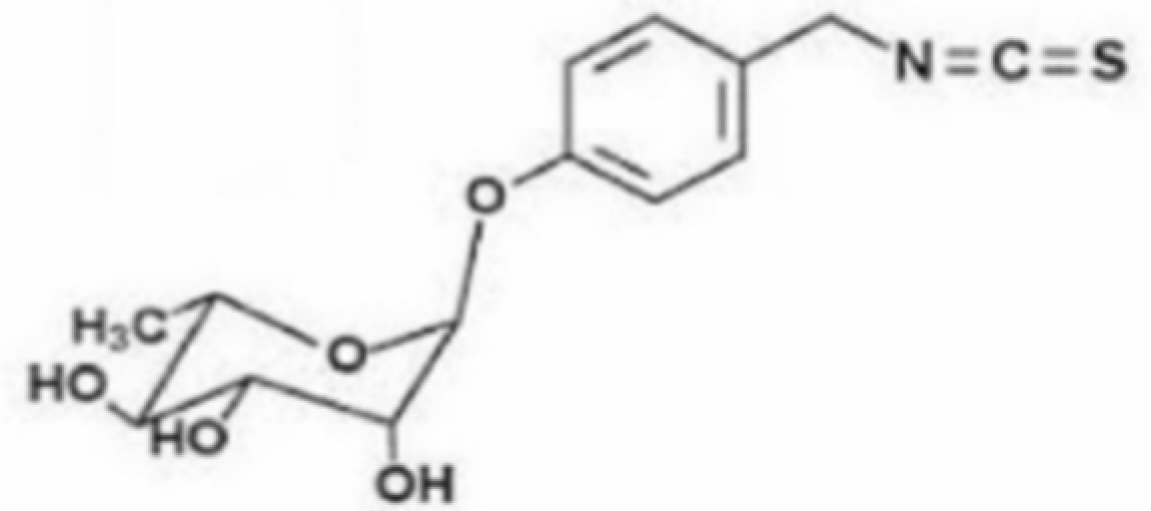	Works against helminthics and inflammation	Abd El‐Hack et al. (2018)

## 5 PROCESSING OF MORINGA

**FIGURE 2:**
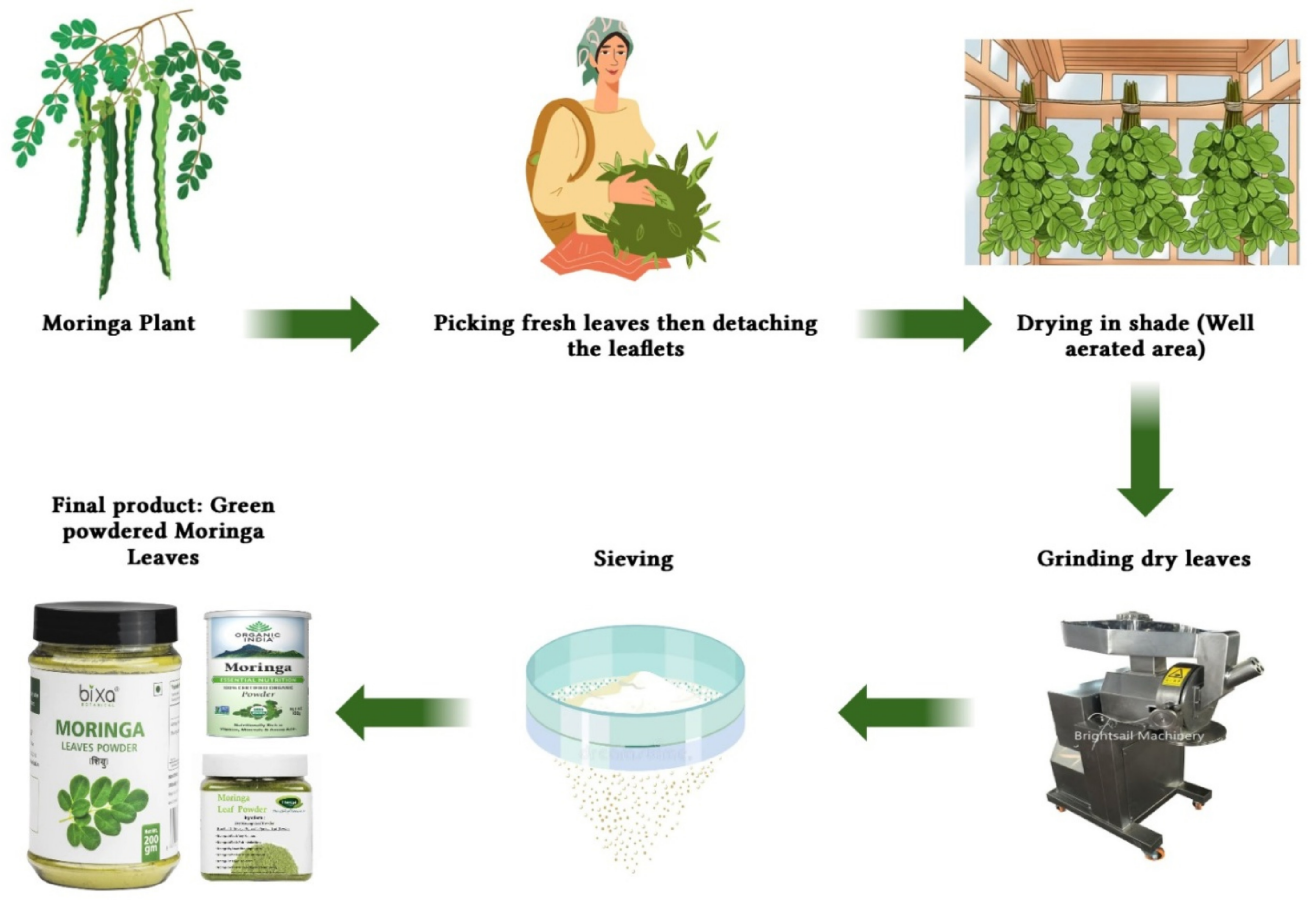
Graphical representation of moringa during processing.

The nutrients in most plants are lost when they are processed. Researchers found that raw and germinated moringa seed flours were higher in phytochemicals, and fermented and germinated moringa seed flours were higher in amino acids (Ijarotimi et al., 2013; Mishra et al., 2012). In germination and fermentation, biochemical and microbial activities can contribute to this. Moringa leaves were studied to see whether nutrients were retained after boiling, simmering, and blanching. There was no difference in cyanide, oxalate, or phytate contents between the two techniques, except that boiling reduced them more significantly than the other two. Plants are usually able to absorb more nutrients from their seeds and leaves when phytate and other antinutrients are present (Kachik et al., 1992; Sallau et al., 2012). Boiling increases iron and antioxidant content, as reported by Yang et al. (2006). It is therefore possible to treat malnutrition problems with moringa seed flour. A slightly bitter taste has been reported in some studies as a reason why children choose not to eat moringa (Nambiar & Parnami, 2008). Moringa noodles are cooked in three different ways—sautéing, steaming, and boiling. Three methods for making noodles were proposed by Kiranawati and Nurjanah (2014). These noodles were studied on rats to see how they affected their mammary glands. Rats' mammary glands produced more milk when sautéed noodles were consumed. Due to the rich insterol content of the oil used, sautéing the noodles improved lactogogum values. There have also been chocolates made from *M. oleifera*. Strengthening cocoa powder with moringa powder was found to be optimal at 20%, according to a study that tested different percentages of moringa inclusion. *Halawa tahinia* contains a similar amount of nutrients as moringa. Researchers have demonstrated that chocolate and *Halawa tahinia* can be developed with high protein and mineral content (Abou‐zaid & Nadir, 2014). To guarantee adequate intake of nutrients in children, there are several moringa fortifications available. Figure 2 depicts the processing of moringa as mentioned below.

### 5.1 Preservation methods

A long shelf life is possible for moringa without a loss of nutrients. You can store the leaves by drying them or freezing them. As reported by Yang et al. (2006), dehydrating the leaves at low temperatures retained more nutrients except vitamin C when compared to freeze dying them. So, to keep nutrients in the leaves at a constant level, you can dry the leaves using household appliances like a stove. Because moringa preserves iron by dehydration, an overdose may cause high iron accumulation. Dehydration does not change the nutritional value of moringa. Hemochromatosis and gastrointestinal distress can occur when iron levels are high. In order to prevent overnutrition, it is recommended to consume 70 g of moringa daily (Asiedu‐Gyekye et al., 2014).

## 6 PHENOLIC COMPOUNDS

**FIGURE 3:**
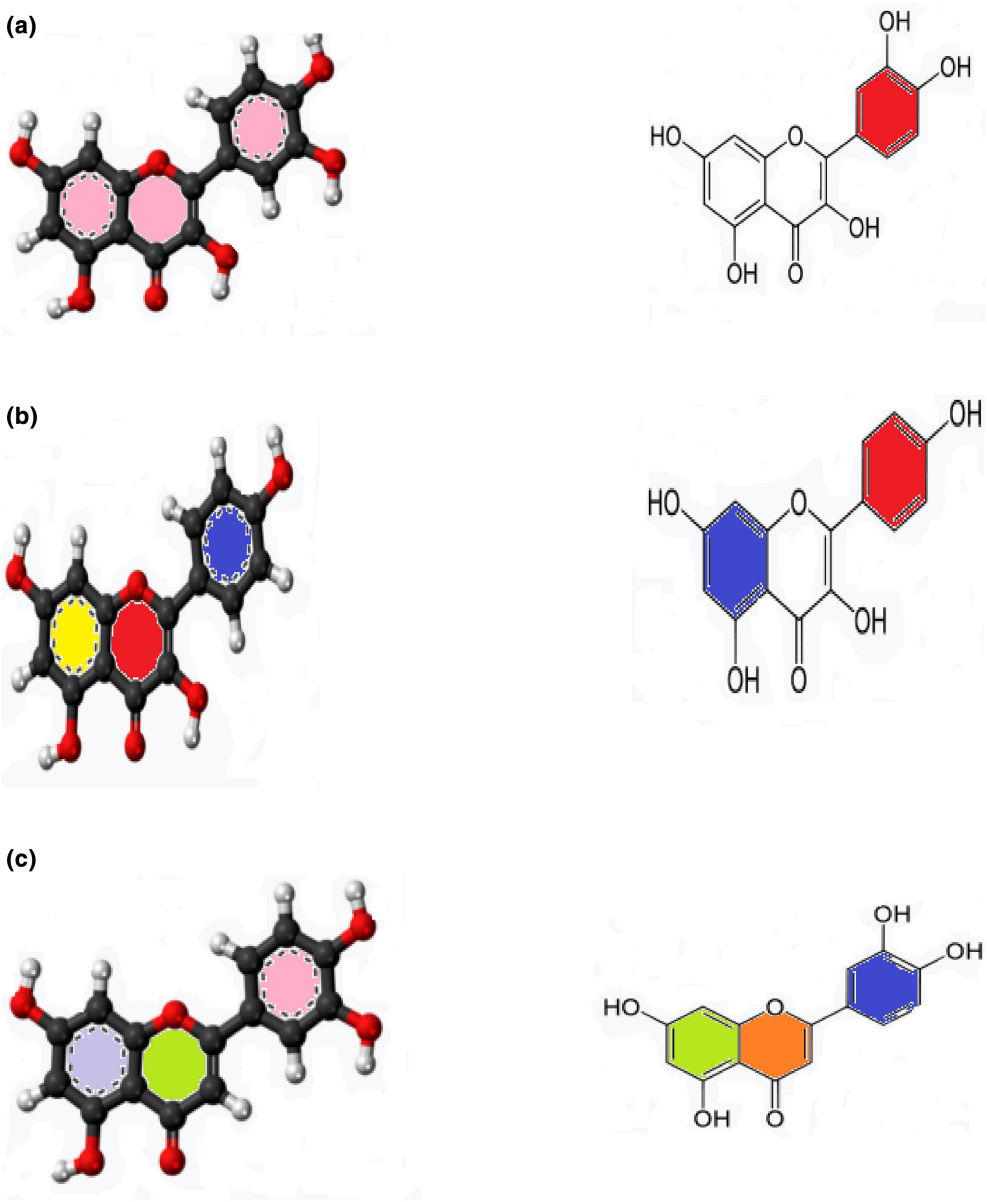
(a) Quercetin. (b) Kaempferol. (c) Luteolin.

The main “free‐phenolics,” also known as phenolic chemicals that are present in plants, are the hydroxybenzoic acid and hydroxycinnamic acid derivatives (bound phenolics). These chemicals which appear in plants as esters or glycosides have one or even more hydroxyl groups attached to the aromatic ring directly (Monera & Maponga, 2012). These hydroxyl groups are what give phenolic compounds their potent scavenging abilities (Mahajan & Mehta, 2009). The moringa plant produced a large number of phenolic compounds, and both in vitro and in vivo studies confirmed their bioactivity. Lignans, kaempferol, including quercetin, apigenin, 26 flavonoids, myricetin luteolin, and 11 phenolic acids and their derivatives, such as feruloylquinic, coumaroylquinic acids, and caffeoylquinic, and their isomers are the main phenolic components found in leaves (Baker et al., 1998; Mahajan & Mehta, 2009). The composition of the methanolic extract from *M. oleifera* was 22% more phenolic in comparison with the young leaves from M, with a total phenolic content that varied between 71.08 to 76.63 mg GAE/g (Al‐Malki & El Rabey, 2015). The leaves of *M. oleifera* are now a more excellent component of these phytonutrients as a result. Many factors, including genotype, growth circumstances, and agroclimatic conditions, affect the phytoconstituents content of moringa plants (Berkovich et al., 2013; Kirisattayakul et al., 2013). In addition to these factors, storage conditions and duration have an effect on the phytochemical content. The bioactive content of plants significantly decreased (from 38% to 53%) and antioxidant capacity declined by 50% at 40°C and 75 5% RH. A foil pouch was used to keep all the samples. Flavonoids were found to make up the majority of the phenolic chemicals detected in moringa leaves (Baker et al., 1998). It is found that leaves contain a number of flavonoids, including quercetin, kaempferol, apigenin, luteolin, and myricetin glycosides. The leaves of the *M. oleifera* plant contain the same levels of quercetin (43.75%) and other flavonoids (18.75%) (Nakamura et al., 2002). It was shown that moringa leaves had higher concentrations of both (1933.7 mg/Kg) and quercetin (1362.6 mg/kg) compared to kaempferol (215.3 mg/kg) and quercetin (17.9 mg/kg) in spinach (Lee & Shacter, 1999; Mishra et al., 2012). Environmental factors have an impact on flavonoids' concentration. With derivatives of hydroxybenzoic and hydroxycinnamic acids, phenolic acids in moringa leaves range from 77 to 187 g per gram DM (Al‐Malki & El Rabey, 2015; Baker et al., 1998; Kirisattayakul et al., 2013). According to a recent study identifying 63 phenolic acids (primarily hydroxycinnamic) in moringa leaves, gallic acid and chlorogenic acid are present in the highest amounts. A number of feruloylquinic acids have been isolated from *M. ovalifolia*, including 4‐acyl, 5‐acyl, p‐coumaroylquinic, caffeoylquinic, as well as trans‐3, 4, and 5 acyl (Oduro et al., 2008). Figure 3a–c shows the chemical structure of different bioactive compounds.

### 6.1 Carotenoids

Photosynthetic plants produce carotenoids as pigments. They are lipophilic substances. They provide protection for the photosynthetic apparatus from harm brought on by excess energy (Leelawat & Leelawat, 2014). Pigment pigments function as antioxidant compounds and provide a number of health benefits, including as defense against cellular aging and degeneration as well as other chronic diseases. They can also be utilized as common dietary additives, like food coloring. Eight different cultivars of *M. oleifera* produce leaves, with fresh weight‐based total carotene concentrations ranging from 44.30 to 80.48 mg/100 g. The six different carotenoids are primarily found in leaves. Total amounts of lutein and carotene in a purified carotenoid extract, antioxidants 2022, 11, and 402 13 showed the highest and lowest purity, with 89% and 94%, respectively (Jung, 2014). The concentration levels of lutein and carotene in fresh leaves are 418 and 272 mg/kg of dry weight, respectively, whereas they are 472 and 166 mg/kg, respectively, in dried powdered leaves (Milla et al., 2021). The amount of carotenes in the leaves is negatively impacted by postharvest circumstances, plant developmental stage, environmental factors, and cooking methods. The highest concentrations of carotenoids are seen in the early phases of plant growth; as plants mature, these concentrations decrease, and postharvest storage at 0°C likewise maintains these concentrations (Chen & Verdes, 2009). Concerns about reducing the prevalence of specific types of cancer are being raised due to the putative antioxidant properties of carotenoids occurrence of specific cancer forms interest in reducing the prevalence of particular cancer kinds.

### 6.2 Alkaloids, glucosinolates, and iso‐thiocyanates

The most harmful secondary metabolites produced by plants are thought to be alkaloids, which contain basic nitrogen atoms. There are four major alkaloids identified in *M. oleifera* leaves, including phenylacetonitrilepyrrolemarumine, 40‐hydroxyphenylethanamide, and rhamnopyranoside (Nair et al., 2005; Oduro et al., 2008). These alkaloids and their derivatives are used to treat a wide range of medical ailments. A glucosinolate from leaves and seeds is the principal member of the glucosinolate class (Asiedu‐Gyekye et al., 2014). A naturally occurring plant enzyme known as myrosinase creates isothiocyanates, nitriles, and thiocarbamates by enzymatic degradation, and these substances are well known for their strong hypotensive and spasmolytic effects (Wright et al., 2006).

### 6.3 Other compounds

The leaves of *M. oleifera* also contain significant amounts of tannins, fatty acids, tannins, and saponins. A crucial water‐soluble vitamin called folate is an essential part of numerous cell metabolisms (Nambiar & Parnami, 2008). The three main forms of folates found in *M. oleifera* are 5‐methyl‐5,6,7,8‐tetrahydrofolic acid, 5‐formyl‐5,6,7,8‐tetrahydrofolic acid, and 10‐formylfolic acid. A rat model investigation discovered that moringa folates had an 81.9% bioavailability, in contrast to the RDA's claim that only 50% of natural folates are bioavailable (Jung, 2014). Due to their greater bioavailability, foods derived from *M. oleifera* and other similar plants are significant sources of folates. Although linoleic acid (6%–13%) and linolenic acid (49%–59%) are the two main polyunsaturated fats, moringa leaves are also a significant source of −3 and −6 polyunsaturated fatty acids. Palmitic acid dominates the saturated fatty acid group, accounting for between 16% and 18% of the total fatty acids present in leaves. *M. oleifera* leaves had higher levels of polyunsaturated fatty acids and lower levels of monounsaturated fatty acids than its pods (Aronson & Rayfield, 2002). Tannins are solvent, unpleasant, polyphenolic macromolecules that precipitate alkaloids, proteins, and other organic substances with a range of 13.2–20.6 g tannins/kg in the dried leaves (Abou‐zaid & Nadir, 2014). The additional substances found in *M. oleifera* leaves are called saponins, and they are made up of an aglycone that is made up of covalently bound sugar moieties and isoprenoid molecules (Rockwood et al., 2013). Per kilogram of dry weight includes 64–81 g of freeze‐dried leaves (Chumark et al., 2008). Together tannin and saponins are believed to provide a number of therapeutic benefits (Kasolo et al., 2010).

## 7 MEDICINAL PROPERTIES

There are more than 300 diseases that can be cured with *M. oleifera*, making it often referred to as a panacea. A traditional herbal medicine used by Indians and Africans is moringa. This medicinal agent is effective because it contains phytochemicals. Reviewing moringa's effects on diseases such as diabetes and cancer is the focus of this section.

### 7.1 Antioxidant properties

By inhibiting the cell's antioxidant defense system, reactive oxygen species oxidize biological molecules, harming DNA, proteins, and carbohydrates, as well as cell membranes. Just a few medical conditions, such as diabetes, heart failure, and hypertension, cause this oxidative stress (Thurber & Fahey, 2010). Consumers always choose natural antioxidants over synthetic ones since they are superior to them (Sutalangka et al., 2013). Beta‐carotene, isothiocyanates, polyphenols, ascorbic acid, rutin, quercetin, and kaempferol are only a few of the potent antioxidants found in *M. oleifera* leaves (Monera & Maponga, 2012; Nair & Varalakshmi, 2011). Many organic solvents, comprising leaf extracts and acetone, chloroform, dichloromethane, diethyl ether, methanol, water, and ethyl acetate, have been found to contain antioxidant properties (Fuglie, 2005; Lalas & Tsaknis, 2002; Mbikay, 2012). The *M. oleifera* ethyl acetate extract can more effectively neutralize the superoxide anion radical (O2), which reduces the interactions of free radicals with biological macromolecules and the subsequent tissue damage (Lalas & Tsaknis, 2002). The development of products that promote the oxidative stability of food items is facilitated by the linear relationship between phenolic compounds' higher antioxidant activity and leaves (see Balakumbahan et al., 2020). Due to the existence of a greater polyphenolic content, the extracts of *M. oleifera* methanolic leaves demonstrated an excellent antioxidant capacity (IC50 49.86 g/mL) in comparison to vitamin C (IC50 56.44 g/mL) (Jung, 2014). Also, it has been claimed that the antioxidant characteristics of leaves are correlated with plants' ability to withstand freezing (Choudhary et al., 2013) and that the compounds found their act as a natural preservative for fat (Sutalangka et al., 2013).

According to a recent study by Khalofah et al. (2020), the application of moringa leaf extract considerably lessened the detrimental effects of cadmium stress on *Lepidium sativum*. In mice affected by aluminum phosphide, 100 mg/kg body mass of moringa extract increases antioxidant levels while lowering malondialdehyde levels (MDA). To reduce the cardiotoxicity brought on by aluminum phosphide (AlP), it might be administered as an adjuvant therapy (Gouda et al., 2018).

Alvarez‐Roman and others' (Álvarez‐Román et al., 2020) topical formulations (nanoparticles and gel) were created using the hydroalcoholic fraction of moringa leaves, and the antioxidant and moisturizing properties of the phytochemical profile were assessed. The formulations exhibited adequate viscosity, pH, and particle size, making them suitable for use as formulations. There were discovered to be seven distinct substances, including phenolic acids and flavonoids. Also, the enhanced antioxidant activity of this formulation and the favorable skin biophysical evaluation outcomes may be leveraged to create a unique approach to administering skin treatments. Goats fed moringa leaves rather than alfalfa hay had improved milk and serum quality, according to a different study (Al‐Juhaimi et al., 2020). Their research looks at three different diets: alfalfa alone, *M. oleifera* leaves at 25%, and goat fodder at 25%. Each study involved 10 goats, a 2‐week adaptation phase, and a 6‐week data collection phase. Goats fed either variety of drumstick leaves exhibited increased content of fat that was free of total phenols and nitrogen extract when compared to goats fed only alfalfa. Saleem et al. (2020) investigated the in silico antioxidant potential of several moringa leaf extracts at doses ranging from 0.1563 to 5 mg/mL. The researchers found that the methanolic extract had the highest DPPH activity at all dosages and that all the extracts showed activity in neutralizing radicals at a small concentration of 0.1563 mg/mL. The methanolic extract also exhibited the highest H_2_O_2_ scavenging activity (70.56, 0.43%) and decreasing power (925.48, 0.45%) at a concentration of 1 mg/mL. Because methanol extracts have larger quantities of total phenolic and flavonoid components than other extracts, it has more antioxidant activity. Aju et al. (Thurber & Fahey, 2010) examined the effects of a methanolic extract from *M. oleifera* on the hearts of diabetic rats under the influence of oxidative stress caused by streptozotocin. During 60 days, rats were given moringa leaves orally at a dose of 300 mg/Kg of body weight. They were divided into six groups: diabetic rats (groups 4 and 5), healthy control rats treated with moringa leaves, healthy control rats fed a high‐energy diet, and healthy control rats given metformin and atorvastatin treatments (groups 4 and 5). Researchers discovered that rats from groups 3 and 4 had much lower concentrations of enzyme activity for antioxidants in their hearts than did rats from groups 2, 5, and 6. The antioxidant activity of moringa leaves is due, among other things, to phytol, DL‐alpha‐tocopherol, hexadonic acid, and other antioxidant substances.

### 7.2 Antimicrobial activity

More and better antimicrobial medications must be created because the incidence of disease‐resistant strains is rising, increasing the number of deaths globally (Kraiczy & Würzner, 2006; Oskay et al., 2009). This is why there has been a rise in the usage of medicinal plants because of their superior health philosophy and lack of adverse drug reactions. *Staphylococcus aureus*, *Escherichia coli*, *Pseudomonas aeruginosa*, *B. subtilus*, *S. typhi*, *P. vulgaris*, *K. pneumonia*, *Micrococcus species*, and *Helicobacter pylori* have all been investigated for their antibacterial efficacy against *M. oleifera* leaf extracts (Abalaka et al., 2012; Ezugwu & Chukwubike, 2014; Kekuda et al., 2010; Patel et al., 2009). In every other circumstance, *P. aeruginosa* exhibits remarkable antibacterial activity; however, the one pathogen that aqueous leaf extract cannot kill is *P. oleifera*. The antibacterial properties of leaves are caused by a variety of phytochemicals (Abalaka et al., 2012). The short peptide 4 (α‐L‐rhamnosyloxy) benzyl‐isothiocyanate was discovered in leaves (Bukar et al., 2010; Suarez et al., 2003), which suggested that it might prevent the growth of microbes by interfering with the formation of the cell membrane or crucial enzymes. 4‐(D‐glucopyranosyl‐1‐4‐Lrhamnopyranosyloxy) benzyl thiocarboxamide, Methyl N‐4‐(lrhamnopyranosyloxy) benzyl carbamate, and 4‐(‐L‐rhamnopyranosyloxy) benzyl glucosinolates are other compounds that give *M. oleifera* leaves their antibacterial properties. *A. niger* has the highest level of inhibition when such antimicrobial effect of steam‐distilled moringa leaves is evaluated against *A. oryzae*, *A. nidulans*, *A. niger*, and *A. terreus* (Abalaka et al., 2012). This is due to the substantial amount of phytochemicals present in the distillate of moringa. The methanolic leaf extract had the best antifungal effectiveness (25 mm) against *Trichoderma harzianum* reported by Ishnava et al. (2012). Leaf extract contains bioactive substances that may function as a natural antibacterial. Moreover, it has been proven that moringa leaves contain pterygospermin, a compound that breaks down into two molecules of the antibacterial benzyl isothiocyanate (Fahey, 2005). The highest zones of inhibition for a 50–50 v/v methanol–water combination were seen for *Salmonella enteritidis* (5.67, 0.47 mm), *Bacillus cereus* (15, 0.00 mm), *Listeria innocua* (10.21, 0.08 mm), and *Salmonella typhimurium* (5.33, 0.47 mm), according to Rocchetti et al. (Miyoshi et al., 2004).

In contrast, *Staphylococcus aureus*, *E. coli*, and *Bacillus subtilis* were all susceptible to the aqueous extract of moringa leaves (MIC: 0.78, 0.78, and 1.56 mg/mL, respectively). Acetone and ethanol extracts from the leaves also showed potent antibacterial activity against these infections. In order to test the antibacterial properties of several solvents, including methanol, water, acetone, ethanol, and ethyl acetate, Prabhakaran et al. (Adeyemi & Elebiyo, 2014) employed a variety of *M. oleifera* plant components, including leaves, flowers, roots, seeds, and bark. *P. aeruginosa* and *E. carotovara* were utilized to test the antibacterial effect using the disc diffusion method. They discovered that leaf extracts in ethanol, methanol, and ethyl acetate inhibited both microorganisms. For the ethanolic leaf extract, the minimum inhibitory concentration (MIC) of 79.3% was excellent. The substantial antibacterial effect is caused by the presence of different phenolics and high total phenolic content. These polyphenols undermine the structural integrity of the cell membrane by interacting with the proteins and enzymes there, lowering cell activity, and finally, killing microorganisms (Mostafa et al., 2018). Higher extract concentrations were shown to dramatically alter the moringa leaves' dose‐dependent antibacterial activity against *Staphylococcus epidermis* (Abadallah & Ali, 2019; Mursyid et al., 2019). The effects of extracts from the leaves of *Matricaria recutita* and *M. oleifera* on 40 susceptible and antibiotic‐resistant bacterial strains were examined by Atef et al. (2019). While plant aqueous and methanol extracts were beneficial to all strains, they discovered that moringa leaf extract had higher levels of activity. The antibacterial activity of deionized and a 95% ethanol extract of *M. oleifera* against pathogens found in water that was polluted was superior to that of other plant parts, according to Bancessi et al. (2020). Another study tested the antibacterial efficacy of moringa leaves against a range of pyogenic bacteria isolated from dromedary camel abscesses using ethanolic and aqueous extracts. They discovered that both extracts effectively warded off each infection. *Corynebacterium ulcerans*, *E. coli*, *Staphylococcus aureus*, *Klebsiella pneumoniae*, *Corynebacterium pseudotuberculosis*, *Proteus vulgaris*, *Pseudomonas aeruginosa*, and *Citrobacter* spp. were all reported to be resistant to the ethanolic extract (Fouad et al., 2019). Due to its larger inhibition zone, this has occurred. An aqueous extract of moringa leaves was successful in removing *Fusarium oxysporum* (9.4, 0.71 mm), *Aspergillus flavous* (12.4, 0.55 mm), *Penicillium italicum* (10.5, 0.26 mm), *Anternaria* sp. (6.6, 0.26 mm), *Aspergillus niger* (15.2, 0.52 mm), and *Rhizopus stolonifera* (13.2, 0.58 mm). Some studies claim that *M. oleifera* leaf extract prevents or avoids the disease's defensive systems, which stops microbial growth and helps to eradicate it (Moyo et al., 2012).

### 7.3 Antidiabetic properties

The moringa plant is capable of curing both type 1 diabetes and type 2 diabetes. The blood glucose level cannot remain within the normal range in patients with type 1 diabetes because they do not produce insulin. Insulin resistance is a characteristic of type 2 diabetes. As a result of beta cells not detecting glucose levels, diabetes type 2 can also be caused by high blood glucose levels, which can be caused by faulty beta cells (Cerf, 2013). There have been several studies demonstrating moringa's antidiabetic properties. *M. oleifera* aqueous extract has been found to cure insulin‐resistant type 2 diabetes and streptozotocin‐induced type 1 diabetes in rats (Divi et al., 2012). Moringa seed powder was fed to rats suffering from STZ‐induced diabetes, and blood glucose levels were reduced after consumption (Al‐Malki & El Rabey, 2015). A dose of 500 mg/kg moringa seed powder also increased serum antioxidant enzymes in rats. Accordingly, moringa's antioxidant compounds may reduce ROS produced in beta cells when STZ is induced (Asiedu‐Gyekye et al., 2014). Beta cells form superoxides and reactive oxygen species (ROS) following ATP dephosphorylation reactions caused by STZ (Wright et al., 2006). High glucose levels in mitochondria cause the release of reactive oxygen species. The low antioxidant content of beta cells leads to apoptosis (Jung, 2014; Sallau et al., 2012). Diabetes mellitus type 2 results from reduced insulin secretion resulting in hyperglycemia. In addition to scavenging ROS, several flavonoids are considered antioxidants. These include quercetin and phenolics. Moringa is hypothesized to contain flavonoids that protect beta cells and control hyperglycemia by scavenging free radicals (Kachik et al., 1992; Rockwood et al., 2013). In addition to retinopathy and nephropathy, diabetes leads to atherosclerosis, among other complications. Such ailments can be prevented with moringa. A high sugar level causes advanced glycated end products (AGEs) when the blood sugar reacts with proteins. Interleukin‐6 and interferons are produced more readily as a result of this interaction. The surface endothelium of arteries has also been shown to express cell adhesion molecules (Aronson & Rayfield, 2002). Atherosclerotic agents can be reduced through the use of moringa (Chumark et al., 2008). Having antioxidant properties, moringa has an antiatherogenic effect.

Diabetes mellitus (DM), a chronic condition brought on by a lack of insulin, a defect in how insulin works, or both, causes delayed hyperglycemia, which in turn affects the body's metabolic functions (Lin et al., 2018; Salim, 2005). It will severely damage tissue and blood vessels, leading to major problems such as retinal, neuropathy, nephropathy, cardiovascular issues, and ulceration (Bearse et al., 2004; Looker et al., 2003; Seki et al., 2004). According to the World Health Organization (WHO), diabetes mellitus affects about 150 million individuals globally. By 2025, the population could surpass 300 million (Omonije et al., 2019). Diabetes comes in two varieties: type 1 and type 2. Type 1 diabetes is characterized by an entire lack of insulin secretion, necessitating the use of insulin replacements (Singab et al., 2014).

Over 90 days of testing, it was discovered that postprandial blood sugar level and glycated hemoglobin had fallen to downtrend of 28.57% and 7.4%, respectively, from their original readings (Momoh et al., 2013). The development and administration of a different concoction including leaf powder, 5% salt, 7% red chili powder, and 7% coriander powder to obese individuals with type 2 diabetes mellitus. Without using any oil, this mixture was heated for a very brief time. They were given 50 g pouches and instructed to eat normally while taking them for 40 days. The produced leaf powder significantly lowered serum blood glucose levels in diabetics (Kumar & Mandapaka, 2013). Low blood sugar levels are caused by antioxidants and blood phenols. Several studies have demonstrated the effectiveness of moringa leaves in treating both forms of diabetes. According to a study on streptozotocin‐induced rats, the crude extract from the leaves of *M. oleifera* may be used for the treatment of type 1 and type 2 mellitus with metabolic syndrome (Khan et al., 2017). Streptozotocin stimulates ATP dephosphorylation, which results in the generation of free radicals and superoxides, with the aid of the beta cells' xanthine oxidase. In addition to killing beta cells, these ROS also stop the release of insulin, which causes hyperglycemia and type 2 diabetes. *Telfairia occidentalis* reported IC50 values of 10.60 g/mL for amylase and 7.69 g/mL for glucosidase, but moringa leaves had much lower IC50 values of 6.49 g/mL for amylase and 4.73 g/mL for glucosidase. Moreover, moringa leaves have increased antioxidant activity levels. Because of their antioxidant characteristics and ability to block both enzymes, moringa leaves have the potential to treat type 2 diabetes mellitus due to their increased phenol content. Methyl isothiocyanate promoted many modifications to the transcriptome and epitome. These pathways mitigate the harmful effects of high glucose levels on renal mesangial cells. Moreover, the DMRs (Differential Methylation Regions) were all 173 and 149 for the categories containing elevated glucose, methyl isothiocyanate, excessive glucose, and reduced glucose, respectively. Methyl isothiocyanate reversed the impacts of the DMRs. These modifications in the pathways have made it simpler to detect the effects of specialized functions for high glucose. The moringa leave's fraction of ethyl acetate was given orally to streptozotocin‐induced diabetic rats for 30 days at a dose of 200 mg/kg body weight. Significant increases were seen in body mass, calorie and hydration intake, blood sugar, insulin, and glycosylated hemoglobin in moringa leaves. Moringa leaves have been shown to be excellent antidiabetic medications because of their higher antioxidant content and reduction of proinflammatory mediators (Bamagous et al., 2018). In a different study, the parotid glands of male albino rats were used to test the antidiabetic effects of moringa leaves. Moringa extract was given orally for 3 weeks at a dose of 200 mg/kg body weight. Rats were administered a huge dose of halothane to put them to sleep following a moringa treatment, and several parotid gland exams were carried out. Much lower levels of blood sugar were achieved. By utilizing optical microscopy, it was possible to observe that acinar cells had begun to grow and take on their original shapes after being destroyed by diabetes, which also caused inner vacuolization and pyknotic nuclei. Compared to control rats, which had many vacuoles and irregular rough endoplasmic reticulum topologies, rats treated with *M. oleifera* had lower vesicles and much more simultaneous cisternae. Rats administered moringa had a much lower tail moment in the comet experiment, demonstrating less damage to DNA (Abo Baker & Moawad, 2020; Leone et al., 2018). Bioactive compounds and high fiber content are the main contributors to the hypoglycemia index. In contrast to secondary metabolites, which are in charge of carbohydrate metabolism and inhibit the enzymes b‐glucosidase and amylase, enhanced amount of fiber slowed down the intestine's glucose absorption and the time it took for the abdomen to empty.

### 7.4 Anticancer properties

There are seven deaths attributed to improper medication each year due to cancer, a common disease. In India, there are 2.4 million cancer cases, but no specific cause has been found for their occurrence. Radiation exposure, smoking, lack of exercise, and lack of exercise are among the factors that cause the disease (Nair et al., 2005). Chemotherapy, radiation, and surgery are expensive treatments that have side effects. In established concentrations, *M. oleifera* is a natural, reliable, and safe anticancer agent. Moringa acts as a potent antiproliferative agent that inhibits cancer cell growth. Leaves are effective as anticancer agents when soluble or solvent extracted. A critical component of cancer's antiproliferative properties may be the release of reactive oxygen species within cancerous cells. Reactive oxygen species induce apoptosis in cells. In addition, caspase 3 and caspase 9 are upregulated in apoptosis, which are important pathways (Jung, 2014; Lee & Shacter, 1999; Tiloke et al., 2013). It is also an excellent anticancer agent because it produces ROS that specifically attacks cancer cells. Tiloke et al. (2013) also demonstrated that the extracts promoted glutathione‐S‐transferase expression with inhibitory effects on antioxidant activity. The use of ROS‐induced anticancer agents is common, but the drugs should also attack antioxidant enzymes (Liou & Storz, 2010). It has, however, been demonstrated that moringa leaf extracts have antioxidant properties as well as anticancer properties that induce ROS. There are still questions to be answered concerning the contradictory characteristics of the leaves. It has yet to be determined exactly how the two opposite attributes of the leaves interact. In addition to glucosinolates and niazimicin, benzyl isothiocyanate in the leaves is also known to have anticancer properties (Hermawan et al., 2012). There are cancer‐related effects associated with benzyl isothiocyanate. The presence of BITC leads to the release of ROS within the cell, which ultimately results in cell death. One reason moringa may be a good anticancer agent is this mechanism (Leelawat & Leelawat, 2014; Miyoshi et al., 2004; Nakamura et al., 2002). One in six deaths is attributed to cancer, which is regarded as a major cause of death globally (McGuire, 2016). Radiation, chemotherapy, and surgery are examples of conventional, often‐used cancer medications and therapies. All of these therapies are expensive and come with a variety of adverse effects. Thus, the scientific community is concentrating on therapeutic plants due to their very powerful phytochemicals. Because its use is safe, natural, and effective when done on a small scale, *M. oleifera* is a potent anticancer agent (Gopalakrishnan et al., 2016). Quercetin, kaempferol (Krishnamurthy et al., 2015), and niazi are substances that can be used as antineoproliferative drugs because they inhibit the growth of cancer cells, according to studies. The potent antiproliferative agents known as ROS, which are created by the moringa extract, attack cancer cells specifically. It was shown that *M. oleifera* leaves stimulated the apoptotic pathway, which prevented HeLa cells from proliferating (Nair & Varalakshmi, 2011). The consequences of *M. oleifera* on the development of pancreatic cancer cells were examined by Berkovich et al. (Jung, 2014). Both the HCT‐8 colorectal cancer cell line and the MDA‐MB‐231 breast cancer cell line saw a considerable increase in apoptosis. In a different study, it was discovered that the extract from moringa leaves increased caspase 3 activity by inducing apoptosis by upregulating BAX expression and downregulating BCL‐2 expression (Balusamy et al., 2019). Two elements contained in leaves, d‐allose and hexadonic acid (palmitic acid), prevent the formation of cancer cells (Yamaguchi et al., 2008). By activating a specific thioredoxin interacting protein (TXNIP) and stabilizing the p27kip1 protein in the G1 phase (G1‐cell cycle arrest), d‐allose inhibits the development of cancer cells while sparing healthy cells (Yamaguchi et al., 2008). HepG2 cells were utilized to examine the anticancer potential of moringa leaf aqueous extracts in humans, who can develop liver cancer. The HepG2 cell growth was greatly decreased (44%–52%) when the leaf extracts were taken internally, making them potent anticancer agents (Jung et al., 2015). According to a different study (Liou & Storz, 2010), the nutritional and physiologically active components in moringa leaves may have a chemoprotective effect, considerably lowering the risk of colorectal cancer brought on by AOM/DSS. Cell death was brought on by the simultaneous overexpression and downregulation of the Bax and Bcl‐2 genes, which was then prevented dose dependently coupled with nuclear modification. Hes‐1 and Notch‐1 levels were reduced by the moringa extract, and the aberrant notch signaling pathway was blocked (Yang et al., 2006). At greater leaf extract concentrations, the ATP and glutathione levels significantly dropped, as demonstrated by the ATP bioluminescence and the ApoGSH colorimetric test. Our research indicates that leaf extract altered the mitochondrial pathway, resulting in cell death. The increase in apoptotic marker expression, a sign of increased cell apoptosis, was confirmed by Western blotting. After 24 h of treatment, a large part of the cells fluoresced, showing active caspase and triggering cell death, according to the FLICA test, which was used to identify cell apoptosis. According to certain reports, moringa leaf extract depolarizes the mitochondrial membrane, which lowers the amount of ATP. The increased ATP level causes ROS and GSH levels to increase, which ultimately leads to cell death. The findings revealed that moringa leaves considerably attenuated DEN‐induced elevations in blood biochemical data and that 8‐OHdG levels in blood dropped by 29%. Hepatocellular tissue's appearance was also improved by moringa leaf therapy. While Bcl‐2, Bcl‐xl, and arrestin‐2 expression were downregulated, Bax and caspase 3 expression increased. This may be due to an increase in ROS formation as well as the potent anticarcinogenic effects of moringa leaves on DEN‐induced hepatocarcinogenesis that promote apoptosis. Another study used a male mouse model to examine the anticancer properties of moringa leaves against AOM/DSS‐induced colon cancer. With a dosage of 5% moringa leaf powder, the activities of tryptophanase, glucosidase, glucuronidase, and urease were all noticeably reduced by 103%, 40%, 43%, and 266%, respectively. Animals exposed to 2.5% and 5% of the chemoprotective moringa leaf powder revealed crypt deformation and reduced adenoma growth. A histological analysis revealed that a higher dose of moringa significantly decreased the risk of malignancies (Liou & Storz, 2010). The mice were given doses of aqueous moringa extract of 200 and 400 mg/kg along with a dose of the antibiotic mitomycin C of 2 mg/kg (MMC). After 50 days with a dose of 400 mg/kg of body weight compared to a dose of 200 mg/kg of body weight, which resulted in a 36.97% drop, the tumor volume was reduced by 78.69%. There was a dose‐dependent tumor shrinking. When doses of moringa were given at rates of 200 mg/kg (12.45, 1.20 g) and 400 mg/kg (8.43, 0.49 g) coupled with 2 mg/kg MMC (14.42, 1.09 g), tumor weight was significantly reduced compared to the control (27.91, 1.50 g). Both cell lines' unfavorable impacts were dose and time dependent, according to in vitro testing. In JB6 cells from the mouse epidermis, methyl isothiocyanate a significant pharmacological component of moringa leaves was tested for its ability to inhibit TPA‐mediated carcinogenesis. Identification of substantially mediated regions (DMRs) and differentially expressed genes was done using DNA methyl seq and RNA seq methods (DERs). The results demonstrated that a number of DMRs and DERs can have their expression reversed by methyl isothiocyanate. A number of inflammatory, Nfr‐2‐mediated antioxidative, and tumor‐suppressive pathways were demonstrated to be restored by methyl isothiocyanate, which was both up‐ and downregulated by TPA (Wang et al., 2019). According to these studies, *M. oleifera* leaves can be used to create nutrient‐rich diets since they contain a number of phytochemicals with potent anticancer properties. As part of their experiment, Sadek et al. (2017) gave Wistar male rats leaf extract (500 mg/kg) for 1 week and leaf extract with Den (10 mg/kg) for 16 weeks has looked into how moringa leaf extract protects against DEN‐induced hepatocellular carcinoma. The results showed that moringa leaves significantly reduced the elevations in blood physicochemical data caused by DEN, and 29% less 8‐OHdG was found. The use of moringa leaf therapy improved the way hepatocellular tissue looked. Bax and caspase 3 expression rose while Bcl‐2, Bcl‐xl, and arrestin‐2 expression was downregulated. This increase in ROS formation may be caused by the powerful anticarcinogenic properties of *M. oleifera* against DEN‐induced hepatocarcinogenesis, which accelerate apoptosis. AOM/DSS‐free mice were divided into four groups, each of which also contained an effective control (10 mg/kg body weight): AOM and three cycles of 5% DSS in group 2, AOM/DSS and 2.5% *M. oleifera* powder in group 3, and AOM and 5% moringa leaf powder in group 3 (group 4). Tryptophanase, glucosidase, glucuronidase, and urease function were all markedly decreased by 103%, 40%, 43%, and 266%, respectively, at a dosage of 5% moringa leaf powder. Mice treated with 2.5% and 5% *M. oleifera* leaf powder, which had a chemopreventive effect, showed crypt distortion and a decrease in adenoma development. A larger dose of moringa reduced the likelihood of tumors by 50%, according to a histological examination (Liou & Storz, 2010).

For in vitro research, human laryngeal carcinoma (Hep‐2) and Ehrlich ascites carcinoma (EAC) cell lines were used, and Balb/c mice were used for in vivo research. Mice were given 2 mg/kg of the antibiotic mitomycin C along with 200 and 400 mg/kg of an aqueous moringa extract. The tumor size decreased dose dependently; after 50 days, the tumor volume fell by 78.69% with a dose of 400 mg/kg of body weight compared to a reduction of 36.97% with a dose of 200 mg/kg. Tumor weight was considerably decreased in comparison to the control when dosages of moringa were administered at rates of 200 mg/kg (12.45, 1.20 g) and 400 mg/kg (8.43, 0.49 g) combined with 2 mg/kg MMC (14.42, 1.09 g) (27.91, 1.50 g). In vitro experiments for both cell lines demonstrated negative impacts that were dose‐and time dependent. Methyl isothiocyanate, a prominent bioactive component of moringa leaves, was examined for its capacity to prevent TPA‐mediated carcinogenesis in JB6 cells from the mouse epidermis. DNA methyl seq and RNA seq technologies were used to identify differentially mediated regions (DMRs) and differentially expressed genes (DERs). The outcomes showed that methyl isothiocyanate can reverse the expression of a number of DMRs and DERs. Methyl isothiocyanate, which was both up‐ and downregulated by TPA, was demonstrated to restore a number of inflammatory, Nfr‐2‐mediated antioxidative, and tumor‐suppressive pathways (Wang et al., 2019). These studies show that the leaves of *M. oleifera* contain a variety of phytochemical substances with strong anticancer capabilities and that they can be used to make nutrient‐rich diets.

### 7.5 Neurosystem activity

**FIGURE 4:**
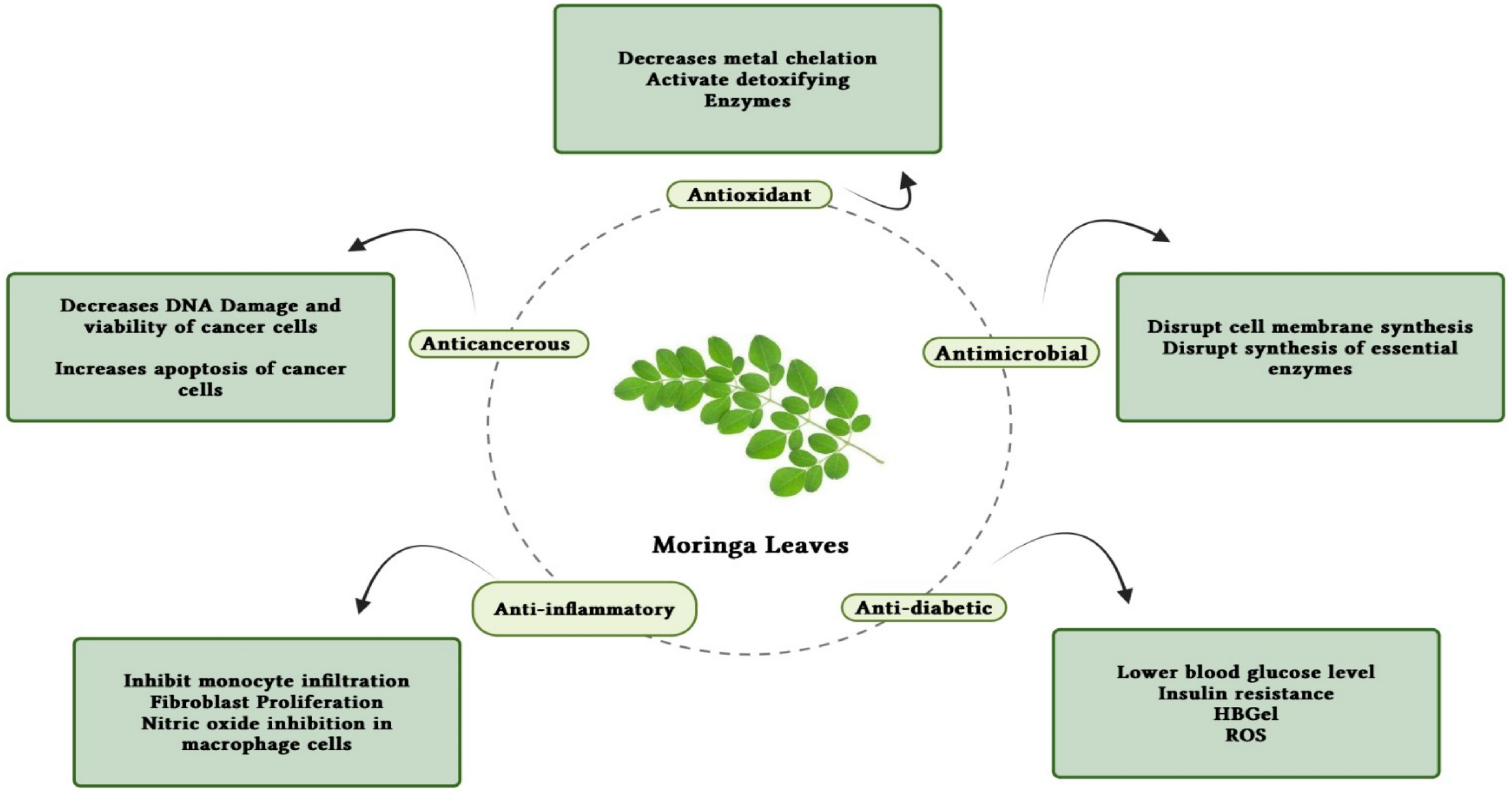
Depicts the moringa leaves with pharmacological properties.

Numerous diseases, such as Huntington's, Parkinson's, epilepsy, Alzheimer's, and other diseases, have an impact on the central nervous system. These conditions affect several body processes, including the ability to concentrate, move, balance, and recall information. Numerous medicinal plants have been seen to treat a wide range of CNS disorders. GABA, which is used to treat neurological diseases like epilepsy and Alzheimer's diseases of the neurological system, was once produced from *M. oleifera* leaves (Bakre et al., 2013). Because of the way it altered hippocampal neurons, it also significantly improved memory. Since moringa therapy raises blood levels of serotonin, which in turn engage the autonomous nervous system to promote deeper sleep, sleep duration is also lengthened (Ray et al., 2004). The impacts of the ethanolic extract of moringa on relaxation of the muscles and locomotor activity were studied by Bhattacharya et al. (2014). They achieved this by using a rotarod test and an actophotometer. Diazepam (10 mg/kg) was given to each of the six rat groups, along with a control dose of normal saline (2 mg/kg) and experimental doses of 50, 100, 200, and 400 mg/kg of moringa extract. The results demonstrated that the drug had a significant impact as a neurological depressant and muscle relaxant. Phytochemicals found in leaves that easily pass the blood–brain barrier and have a stimulatory impact on the GABA receptor complex may be the cause of this activity. Al‐Abri et al. (2018) also looked into how a moringa leaf extract affected the motor and behavioral responses of mice. The control group was given an oral dose of 0.9% brine during the treatment group's 14‐day treatment period, while the latter got 100, 200, and 400 mg/kg of a water‐soluble extract of moringa leaves. The antinociceptive activity was found to be strongly dose dependent in both chemical and thermal testing. During the forced swimming test, mice given the highest dose (400 mg/kg) displayed lower exploratory function, neuromuscular coordination, and movement time, but there were no obvious variations in motor activity between dosages. Moringa leaves were studied by Mahaman et al. (2018) in rat models of Alzheimer's disease brought on by hyperhomocysteinemia (HHcy). Rats were given 400 g/kg/day of HHcy through the vena caudalis for 14 days. The rats were given a methanolic extract of moringa at doses of 200 and 400 mg/kg/day as a prophylactic and therapeutic therapy. As a positive control, SCR1693 (1 mg/kg/day) was administered. Neurodegeneration was slowed as synaptic proteins were repaired by moringa treatment. A crucial stage in this process, the downregulation of calpain activity by moringa leaves, reduced the pathophysiology brought on by HHcy‐induced tau phosphorylation and A. The authors thus offer fresh perspectives on the form of Alzheimer's disease that is currently incurable. The main contributor to brain dysfunction is oxidative stress, and a moringa leaf ethanolic extract significantly lessened the neurotoxic consequences of CoCl2‐induced hypoxia. The 50 male rats were assigned to 5 groups of 10 each. Group 1 served as the control group, while group 2 was given an oral dose of a 400 mg/kg moringa ethanolic extract, group 3 was given an oral dose of cobalt (II) chloride (CoCl2) at a concentration of 40 mg/kg for 60 days, group 4 was given extract for 15 days before and during CoCl2 treatment, and group 5 was given extract for 15 days after cobalt (II) chloride treatment. Monoamine and GABA neurotransmitter levels were significantly reduced in hypoxic rats. Moreover, the fraction of neurons with a GFAP‐positive astroglia score rose, and redox signaling gene expression was changed. When moringa extract was given before and at the same time as CoCl2, positive neurotoxic effects were seen (Mohamed et al., 2019). The neuroprotective properties of an ethanolic extract of moringa leaves were also investigated in rats receiving quinine therapy, specifically in the myelin and cerebellar neurofibers. The control group was group 1, while quinine doses of 10, 20, and 30 mg/kg were administered to groups 2–4, respectively. Groups 5, 6, and 7 each received 10 mg/kg quinine and 250 mg/kg leaf extract, 20 mg/kg quinine and 500 mg/kg leaf extract, and 30 mg/kg quinine and 750 mg/kg leaf extract, respectively. Every treatment lasted 7 days. The Figure 4 presents the pharmacological properties of the drumstick plant. The findings showed that rats in group 5 revealed full neural protection, neuronal regeneration, and regular repair of cerebellar cytoarchitecture, in contrast to groups 6 and 7, which showed only minor structural damage to the cerebellum. The leaf extract's neuroprotective impacts on the central nervous system were predominantly brought about by flavonoids (Umoh et al., 2018).

### 7.6 Other diseases

An important neuroprotective component of moringa is its antioxidant properties. When blood flow to the brain is restricted, cerebral ischemia occurs. As a result, lipoperoxidation and reperfusion occur, resulting in reactive oxygen species. Because it contains antioxidants, moringa contributes to the protection of the brain (Baker et al., 1998; Kirisattayakul et al., 2013). There has been evidence that *M. oleifera* promotes spatial memory, making it useful in treating dementia. Several studies have shown that the leaf extracts help improve cholinergic function and memory by decreasing the enzyme acetylcholine esterase activity (Piskula et al., 1999). As reported by Adeyemi and Elebiyo (2014), moringa reduces urea and creatinine levels in rats' blood and prevents renal dysfunction. Using doses of 500 mg and 350 mg, moringa was shown to possess antiulcer properties. 86.15% and 85.13% of acidity were reduced in gastric ulcers, respectively (Chinma et al., 2014). Patients with AIDS can benefit from moringa if they receive prescriptions from herbal practitioners. Including moringa in a HIV‐positive individual's diet may boost their immune system. To validate moringa's effects on antiretrovirals, more research is necessary (Monera & Maponga, 2012). Rats with arthritis were treated with moringa flower extracts that significantly reduced rheumatoid factor, TNF‐alpha, and IL‐1 levels. There are numerous studies to support the claim that moringa is an effective treatment for arthritis (Mahajan & Mehta, 2009). Antimicrobial agents are needed to counter microbial diseases, and *M. oleifera* has proven to be an effective one (Chen & Verdes, 2009). *Vibrio cholera* and *Bacillus subtilis* are among the bacteria that are resistant to *M. oleifera* extracts. Seeds contained pterygospermin, moringine, and benzyl isothiocyanate were found to have antibacterial properties (Sutalangka et al., 2013) (Table 4).

**TABLE 4:** Health benefits, sample type, and model type with summary.

Health benefits	Sample type	Model type	Result summary/mechanisms	References
Antimicrobial	Aqueous leaf extract	Agar diffusion method	Inhibited the growth of *E. coli*, *S. typhi*, and *P. aeruginosa* Minimum inhibitory concentration (MIC): 10–20 mg/mL Significant variation in antimicrobial effectiveness (MIC for bacteria: 0.04–2.50 mg/mL and MIC for fungi: 0.16 to >2.50 mg/mL) Coefficient of variability for bacteria during winter (75.2%) and summer (31.3%) Coefficient of variability for fungi during winter (19.2%) and summer (23.1%) Samples collected in winter exhibited greater antifungal activity Minimum bactericidal concentration (MBC): 20–40 mg/mL Inhibited the growth of certain bacterial strains	Mostafa et al. (2018)
	Acetone extract from 12 moringa trees harvested in different seasons	Twofold serial dilution method		Mursyid et al. (2019)
	Different extract of moringa leaves	Well‐diffusion assay		Baker et al. (1998)
Anticancerous	Moringa leaves powder	Colorectal carcinogenesis model (24 male mice)	Suppressed colorectal carcinogenesis induced by AOM/DSS with a 5% w/v dosage of moringa The ethanolic extract impedes the growth of C4‐II and HeLa cervical cancer cells by reducing NF‐kB and Bcl‐xL levels in these cells Moringa leaves collaborate with vesicular stomatitis virus for cervical cancer therapy by modifying pathways related to proliferation, apoptosis, and antiviral responses Moringa leaves elevated BCL‐2 expression in liver and kidney tissues, leading to reduced levels of caspase 3, caspase 9, and NKFβ markers	Abadallah and Ali (2019)
	Different extract of moringa leaves	Cervical cancer cell lines		Atef et al. (2019)
	Methanolic extract	48 male Wistar rats		Bancessi et al. (2020)
Antioxidant	Subcritical ethanolic leaves extract of flavonoids	DPPH and FRAP assay	FRAP assay = 0.95–1.35 mmol FeSO4/mg DPPH assay (IC50 value) = 0.7440 mg/L	Al‐Juhaimi et al. (2020)
Antidiabetic	Aqueous leaf extract	Albino rats	A dose of 300 mg/kg resulted in a decrease of 33.18% and 44.06% in blood sugar levels in normoglycemic and hyperglycemic rats after 6 h Daily administration of 8 g leaf powder for 40 days led to a reduction of 28% in fasting plasma glucose (FPG) and 26% in postprandial blood glucose (PPPG)	Bancessi et al. (2020)
	Moringa leaves powder	Untreated type 2 diabetic patients (30–60 years of age)		Fouad et al. (2019)

## 8 BIOAVAILABILITY AND BIOACCESSIBILITY

Different recent studies examined the nutritive as well as pharmacological profiles of food matrices and by‐products, but it is more important to comprehend their bioaccessibility and bioavailability than to determine the precise quantity of certain compounds. Contrary to bioavailability, which indicates how much of the compounds that have been digested are normally absorbed and metabolized, bioaccessibility describes how many polyphenols are liberated for absorption from the food material in the digestive system (Žugˇci'c et al., 2019). According to a study, free polyphenols including caeffic, morin, gallic, and kaempferol, as well as mono‐ and oligosaccharides like mannose and stachyose, have significant bioaccessibility (6–210). Although p‐coumaric acid and quercetin showed more efficacy, gallic acid, vanillin, chlorogenic acid, and rutin all exhibited increased bioaccessibility at the stomach level (Caicedo‐Lopez et al., 2019). Bioavailability and bioaccessibility of different bioactive compounds are mentioned in Table 5. According to Dou et al. (2019), 2.48 (phenolics) and 2.20 (flavonoids) times their initial quantities were released throughout the entire digestive process. The amount of phenolics and flavonoids produced during oral digestion was higher, at 49.6% and 58.4%, respectively, but the amount created during stomach digestion was lower. Due to the fast breakdown of flavonoids by digestive enzymes, the small intestine contained more phenolic acids than flavonoids. 6,8‐Di‐C‐glucosylapigenin, catechin, ferulic acid, and quercetin‐3‐O‐D‐glucoside were the main phenolic substances produced during oral, gastric, and intestinal digestion, respectively. The glycosylated flavonoids apigenin, quercetin, and kaempferol are among those found in moringa leaves. According to Crespy et al. (2002), several flavonoids in their glycosidic forms could not be absorbed by the stomachs of Wistar rats, including quercetin. In a different investigation, the rats' stomachs quickly absorbed isoflavone aglycones and their blood plasma comprised metabolite. When utilized as a tiny absorption site in the stomach, isoflavone glucosides did not have the same impacts (Piskula et al., 1999). Rutin, which was metabolized by humans and rats slower than quercetin (Crespy et al., 2002; Tesfay et al., 2017), showed another effect of glycosylation. Because of the high levels of phytic acid in moringa, iron is not bioavailable at all (Hollman et al., 1999; Manach et al., 1997). In moringa leaves, 5,6,7,8, 5‐formyl‐5,6,7,8, 5‐methyl‐5,6,7,8, and 10‐formylfolic acid are the main types of folate that may be present (Gallaher et al., 2017). When compared to other green vegetables, moringa leaves contain a high biological value of several different forms of folate. In rat models, *M. oleifera* folates were found to have an 81.9% higher bioavailability than synthetic folates (Saini et al., 2016). The stomach's susceptibility to methylation, absorption, fermentation, and digestion can also differ. Extensive in vitro and in vivo research is needed to understand how the polyphenols in moringa leaves are absorbed and used metabolically. Functional meals are essential to modern living since they help avoid many chronic illnesses or lessen their severity. Functional foods have been the subject of much research, and it has been discovered that because they support health, they are essential for meeting consumer expectations (Ayala‐Zavala et al., 2011). A great deal of chronic disorders can be brought on by the body's overproduction of free radicals, which can also cause the body to break down important macromolecules like DNA, lipids, and proteins (Serafini et al., 2006). Antioxidants may therefore be extremely important in the treatment of chronic illnesses. *Moringa oleifera* is becoming known as a crucial functional food due to the improved nutritional value of its edible portions and the presence of powerful antioxidant components (Figure 3a–c).

**TABLE 5:** Bioavailability and bioaccessibility of bioactive compounds in *Moringa oleifera.*

S. No.	Compound	Bio accessibility/bioavailability	Remarks	References
1	Mono‐/oligosaccharides (mannose and stachyose)	High bioaccessibility (6%–210%)	Mono‐/oligosaccharides and antioxidants in moringa leaves showed high bioaccessibility	Rodriguez (2021)
2	Antioxidants in Moringa Leaves	High bioaccessibility (6%–210%)	‐	
3	P‐Coumaric Acid	Higher value in small intestine stage	P‐coumaric acid showed higher bioaccessibility in the small intestine stage	
4	Quercetin	Higher value in small intestine stage	‐	
5	Gallic Acid, Chlorogenic Acid, Vanillin, and Rutin	Better bioaccessibility at stomach level	These compounds exhibited better bioaccessibility at the stomach level	Goordeen and Mohammed (2021)
6	Flavonoids and Phenolics	Released 2.48 and 2.20 times, respectively, following full digestion	Phenolics and flavonoids were released following full digestion. Largest amounts released during oral digestion. Gastric digestion released less than oral digestion	
7	Phenolic Acids	More than flavonoids in small intestine	Phenolic acids were more abundant than flavonoids in the small intestine due to flavonoid breakdown by digestive enzymes	
8	Main Phenolic Substances After Digestion	6, 8‐di‐C‐glucosylapigenin, Catechin, Ferulic Acid, Quercetin‐3‐O‐D‐glucoside	Produced after oral, gastric, and intestinal digestion, respectively	Gokulapriya et al. (2022)
9	Flavonoids in Moringa Leaves	Mostly glycosylated	Majority of flavonoids occur in glycosylated form	
10	Folic Acid Types in Moringa Leaves	High bioavailability compared to other green vegetables	Moringa leaves have high bioavailability of these folate types compared to other green vegetables	
11	*Moringa oleifera* Folate versus Synthetic Folate	81.9% higher bioavailability in rat models	*Moringa oleifera* folates had 81.9% higher bioavailability compared to synthetic folates in rat models	

## 9 USE OF MORINGA LEAVES IN FOOD ITEMS FOR NUTRITION PURPOSES

Moringa leaves can be used as nutraceuticals to address the difficulties of global malnutrition because of their high level of nutrients and built‐in antioxidants (Barminas et al., 1998; Kiranawati & Nurjanah, 2014). Recent research (Gokulapriya et al., 2022; Goordeen & Mohammed, 2021) indicates that moringa leaves are extensively used in the preparation of nutritious foods. The nutritious value of several baked goods was significantly increased by the application of moringa leaves. The proteins and crude fiber contents of wheat flour bread significantly increased to 54% and 56%, respectively, when the bread was fortified with 5% moringa leaves. However, other studies found that the increase in crude fiber in bread that had been fortified was higher (88%) than the increase in protein (17%). The amount of fat and protein in tortilla chips was enhanced by 50% by the introduction of 1%, 3%, and 5% moringa leaf flour (Shah et al., 2015). The bulk of the fatty acids in the improved tortilla chips were oleic and linoleic acids. The inclusion of moringa leaves dramatically boosted the TPC and antioxidant properties (Olusanya et al., 2020). Fombang and Saa (Sengev et al., 2013) report that functional tea prepared from drumstick leaves has a high level of phenolics and 81% reduction in the DPPH test when specimens are processed for 35 min at 97°C with a solid‐to‐liquid ratio of 1/20 mg/mL. Moreover, successful results have been obtained from investigations on microbial food preservation using fermented foods and moringa leaves. When applied to an edible coating formulation for avocados, moringa seed and leaf extraction have been shown to have antifungal properties toward *C. gloeosporioides*, *A. alternate*, and *L. theobromae* strains (Páramo‐Calderón et al., 2019). More inhibition was present in the ethanolic leaf extract than the methanolic extract. Fruits also produced less ethylene and breathed more slowly. These results are directly attributable to the moringa leaves' greater phenol content. The amount of fat, fiber, and minerals in mahewu was significantly increased by the inclusion of moringa leaves. With 2%, 4%, and 6% more moringa leaves, respectively, the amounts of calcium (106%, 214%, and 287%) and iron (350%, 700%, and 900%) were increased. The most delicious drinks had a 2% fortification (Fombang & Saa, 2016). The high nutritional content of moringa, which includes antioxidant and protein components made up of polyphenols and vitamins, is largely responsible for its health advantages. Moringa leaves can be utilized as functional foods and are essential for a balanced diet because of these factors.

## 10 CONCLUSION AND FUTURE PROSPECTS

Indian researchers have yet to gain acclaim for *M. oleifera* research. A variety of purposes can be served by utilizing the nutrients found in this wonder tree. A great deal of anticancer and antidiabetic properties are associated with *M. oleifera*. Moringa's health benefits, however, are less well documented in double‐blind studies. The primary mechanisms by which moringa works as an antidiabetic and an anticancer agent need to be further examined. Aqueous extracts may have antioxidant properties against cancer cells, but further research is needed. It has also been noted that aqueous extracts contain antioxidants. Irony cannot be fully understood until the exact mechanism is understood. Further research is needed to understand how environmental factors influence nutrient levels in *M. oleifera* globally, this plant is grown for its leaves and other parts. In the future, it may be possible to develop novel therapeutic compounds from *M. oleifera* by isolating endophytic fungi and identifying enzymes or proteins responsible for their anticancer and antidiabetic activity. *M. oleifera* can also be evaluated as a biocoagulant in the context of commercial use. A viable water purification alternative could be found in it. In the market, there is a huge demand for snacks. Thus, moringa‐fortified snacks are a double win to eradicate malnutrition. Research can contribute to the nation's income by confirming earlier studies regarding the tree's potential to provide highly nutritious foods.

## AUTHOR CONTRIBUTIONS

**Zarina:** Writing – original draft (equal). **Monisha Rawat:** Formal analysis (equal); writing – original draft (equal). **Harjinder Kaur:** Formal analysis (equal); writing – original draft (equal). **Sachitanand Das:** Conceptualization (equal); formal analysis (equal). **Taranpreet Kaur:** Investigation (equal); visualization (equal). **Noor Akram:** Writing – review and editing (equal). **Syed Saad Jan:** Visualization (equal). **Nabila Nusrat Oyshe:** Editing, Writing, Validation (equal). **Zargham Faisal:** Review and editing. **Yasir Abbas Shah:** Validation, Visualization. **Mahbubur Rahman Khan:** Revising, Reviewing, Editing. **Ab Waheed Wani:** Original draft.

## CONFLICT OF INTEREST STATEMENT

The authors declare no conflicts of interest.

## Data Availability

Even though adequate data have been given in the form of tables and figures, all authors declare that if more data are required then the data will be provided on a request basis.
